# Prioritization of solid concentration and temperature for solid state anaerobic digestion of pearl millet straw employing multi-criteria assessment tool

**DOI:** 10.1038/s41598-019-48437-1

**Published:** 2019-08-15

**Authors:** Kunwar Paritosh, Nidhi Pareek, Aakash Chawade, Vivekanand Vivekanand

**Affiliations:** 1 Centre for Energy and Environment, Malaviya National Institute of Technology Jaipur, Jaipur, Rajasthan 302 017 India; 2 Department of Microbiology, School of Life Sciences, Central University of Rajasthan Bandarsindri, Kishangarh, Ajmer, Rajasthan 305 801 India; 3 Department of Plant Breeding, Swedish University of Agricultural Sciences, P.O. Box 101, 230 53 Alnarp, Sweden

**Keywords:** Environmental biotechnology, Biogas

## Abstract

India produces huge quantities of agricultural residues and stubbles and mainly disposed by burning on site causing air pollution. The organic matter present in the residues and stubble may be utilized by anaerobic digestion as a source of renewable energy subsequently reducing emission of greenhouse gases caused by burning. In the present study, solid state anaerobic digestion (SSAD) of pearl millet straw was investigated at mesophilic and thermophilic temperature with four different total solid (TS) content (15, 20, 25 and 30%). Results showed that 20 and 25% TS generated maximum methane (124.1 ± 7 and 162.4 ± 9L/kg VS) at mesophilic and thermophilic temperature respectively. However, increasing TS content beyond 25% did not show significant increment on methane yield. Analytical analysis showed correlation between the reduction of volatile solids and methane yield as well as VFA (volatile fatty acid) accumulation at high TS content. Also, VlseKriterijuska Optimizacija Komoromisno Resenje (VIKOR) and Technique for Order Preference by Similarity to Ideal Solution (TOPSIS) methods as MultiCriteria Decision Making modelling (MCDM) applied to select best possible alternative for SSAD of pearl millet. MCDM analysis showed that VIKOR method endorsed the experimental results.

## Introduction

Lignocellulosic residues and stubble are promising alternatives to provide fuel and energy security by utilizing them as a source of biomethane production^1,2^. These resources have mainly 9–80% cellulose followed by 10–50% hemicellulose and lignin accounts for 5–35% of the lignocellulosic residues. Cellulose and hemicellulose are digestible components while lignin shows recalcitrant nature in the anaerobic digestion process^1^. Biogas generated by digesting lignocellulosic residues anaerobically may be utilized directly in combined heat and power (CHP) units, cooking or may be purified for transportation purposes. This will reduce the ongoing burden on fossil fuels and eventually helps to mitigate greenhouse gases (GHG)^3^. In general, anaerobic digestion (AD) for biomethane production may be classified into two categories based on TS concentration, liquid state anaerobic digestion (L – AD; <15% TS) and SSAD (>15% TS). As per literature, SSAD systems can be fruitful in terms of loading rate of the feedstocks or organic loading rate (OLR), may decrease the volume of the reactor, lowering the heating demand if required and could provide higher volumetric biomethane production^4^ (Table 1). In a study performed by Brown *et al*.^5^, SSAD of switchgrass and corn stover showed almost equal volumetric productivity and the volume of SSAD reactor (1L) was considerably less i.e. 50% to that in which LAD (2L) was performed. Recent research and development in SSAD have attracted many researchers in the past decade and around 60% of recently built AD system has adopted SSAD system^4^. Besides having numerous benefits of SSAD process and progress in system designs, there are many aspects that need to be improved for further technical development, scale-up and commercialization of the technology. The retention time of SSAD has been documented to be up to three times longer than liquid AD as the mass transfer rate is slow in SSAD systems than that of L-AD.

**Table 1:** Comparison of solid and liquid state anaerobic digestion.

Parameters	LAD	SSAD
Feedstock	Sewage sludge, Wastewater, Liquid manure	Organic fraction of municipal solid waste, lignocellulosic biomass, agricultural residues
Total solid	<15%	>15%
Organic loading rate	2–5 kg VS/m^3^	5–12 kg VS/m^3^
Abrasion of reactor	Sand and grit may cause abrasion	Not prone to abrasion because of sand and grit
Effluent	Large volume of effluent, not very easy to handle.	Handling of effluent is comparatively.
Operation	Mixing is required. Short circuiting may happen	Moving parts are limited which ensures less operational problems
Commercial suppliers around the globe	BTA, KCA, BIOSTAB, WAASA	DRANCO, Kompogas, Valorga, Biocel, BRV

The key factors which drive the SSAD reactors are TS and temperature condition (mesophilic, 35 °C; thermophilic, 55 °C). While TS content is responsible for the mass transfer in the SSAD reactors, temperature condition determines the fate of the microbes in the reactor which may disturb the overall reaction process^6^. Solid state mesophilic specific methanogenic activity was observed by Hyaric *et al*.^7^, by employing municipal solid waste. Four moisture contents were selected ranging from 65 to 82%. Results revealed that solid concentration affects the specific methanogenic activity in SSAD reactor. Also, the results showed a linear relationship between the specific methanogenic activity and solid concentration. Palm oil mill industry waste such as empty fruit bunches, oil palm fronds and oil palm trunks were digested anaerobically for biomethane yield under three different solid concentrations (16, 25 and 35%)^8^. Result of the trials showed that at 16% TS, all the reactor fed with various substrates had higher methane yield followed by 25 and 35% TS in the reactors. The reason was ascribed to the fact that increased TS content hindered the gas liquid-transfer causing the accumulation of CO_2_ and H_2_. It was concluded that the total solid removal efficiency was also better in the reactor with 16% TS^8^.

Thermophilic and mesophilic temperature conditions have been widely adopted for the AD of organic municipal waste and lignocellulosic biomass residues. The mesophilic temperature range may be stable to that of thermophilic temperature conditions and helps in kickstarting the digestion process in AD systems which is quite easy in thermophilic zone as it accelerates the hydrolysis process of influent^9^. Effect of ammonia – N accumulation in SSAD process of food waste, fruit and vegetable waste, yard waste and paper waste was performed by Zeshan *et al*.^10^ using the pilot scale thermophilic reactor for the same and results showed that the net energy gain was around 50 to 75% higher in the thermophilic temperature condition to that of mesophilic one. Although thermophilic SSAD increases the methane and biogas productivity, it also requires heat energy input for the uninterrupted process and net energy gain may be less as compared to mesophilic condition. Sheets *et al*.^11^ observed that besides having a higher production rate of methane, lower net energy gain was achieved in thermophilic condition while digesting switchgrass anaerobically. Also, thermophilic condition in SSAD enhances the hydrolysis of the substrate by stimulating hydrolytic microorganisms in the reactor. This acceleration of hydrolysis in the SSAD reactor may cause a rapid increase and accumulation of VFAs in the reactor^12^ that may hamper or inhibit the methanogenesis process of the bioreactor. In this regard, TS and carbon to nitrogen ratio of substrate(s) will have a noteworthy role in the digestion process^13^.

Multi Criteria Decision Making models (MCDM) is normally applied for both indefinite and definite set of scenarios. SSAD of pearl millet straw (PMS) at mesophilic and thermophilic temperature is a definite set of scenarios having a definite set of output. For definite set of scenarios, there are many MCDM techniques such as ELECTRE (elimination et choix traduisant la realité), PROMETHEE (preference ranking organization method of enrichment evaluation), TOPSIS (technique for order preference by similarity to ideal solution) and VIKOR (VlseKriterijuska Optimizacija I Komoromisno Resenje)^14^. Few previously reported studies have applied VIKOR and TOPSIS techniques in the field of renewable and sustainable energy for the selection of best possible outcome^15,16^.

To the best of our knowledge, no reports are available where SSAD of PMS has been attempted for enhanced biogas production and application of multicriteria decision making model (MCDM) for SSAD to have best possible alternative. PMS is widely available in the north-west part of India and gross production is around 24 MT per year. The objective of the present study was to study the effect of TS content of PMS at thermophilic and mesophilic temperature in a solid state anaerobic medium for biogas production and applying MCDM to select the best output considering multiple output parameters of AD such as pH, organic matter removal, alkalinity and volatile fatty acid along with methane yield.

## Results and Discussion

### Composition of feedstock and inoculum

The raw PMS used for this study had long (6 cm) and dust free stalk which was intact. Before study, PMS was shredded with the help of scissor to the length of 0.5 to 1 cm for batch biochemical methane potential (BMP) test. PMS had high TS (93.42%) and VS (92.24% of TS) content and considerable amount of cellulose (36.42%) and hemicellulose (25.31%) with moderate lignin content (15.63%) while VS in inoculum was 65.78% VS (% TS) (Table 2) which shows its suitability for SSAD for biogas production. It was reported that high TS content in feedstock may cause inhibition in hydrolysis step of AD, accumulated VFAs in the reactor and limited nitrogen supply which may reduce the overall methanogenesis in SSAD^12^. In the present study, PMS and inoculum had C/N ratios of 48.87 and 20.12 respectively. Moreover, the nitrogen content in inoculum was observed to be more than 2 fold to that of PMS which may help to subside the effect of limited supply of nitrogen because of high TS content in SSAD^17^.

**Table 2:** Characteristics of pearl millet straw and inoculum.

Parameters	Pearl millet straw	Inoculum
TS (% DW)	93.42 ± 2.8	7.51 ± 0.3
VS (% TS)	92.24 ± 1.9	65.78 ± 0.1
C (% DW)	39.59 ± 2.2	35.21 ± 0.1
H (% DW)	7.44 ± 1.4	4.34 ± 0.9
N (% DW)	0.81 v 0.1	1.75 ± 0.2
O (% DW)	47.82 ± 1.7	57.87 ± 1.1
C/N	48.87 ± 1.6	20.12 ± 0.1
Cellulose (% DW)	36.42 ± 2.5	ND
Hemicellulose (% DW)	25.31 ± 1.8	ND
Lignin (% DW)	15.63 ± 0.6	ND

VS – Volatile solid; DW – dry weight; ND – not determined.

### Effect of TS and temperature on SSAD performance

**Figure 1:**
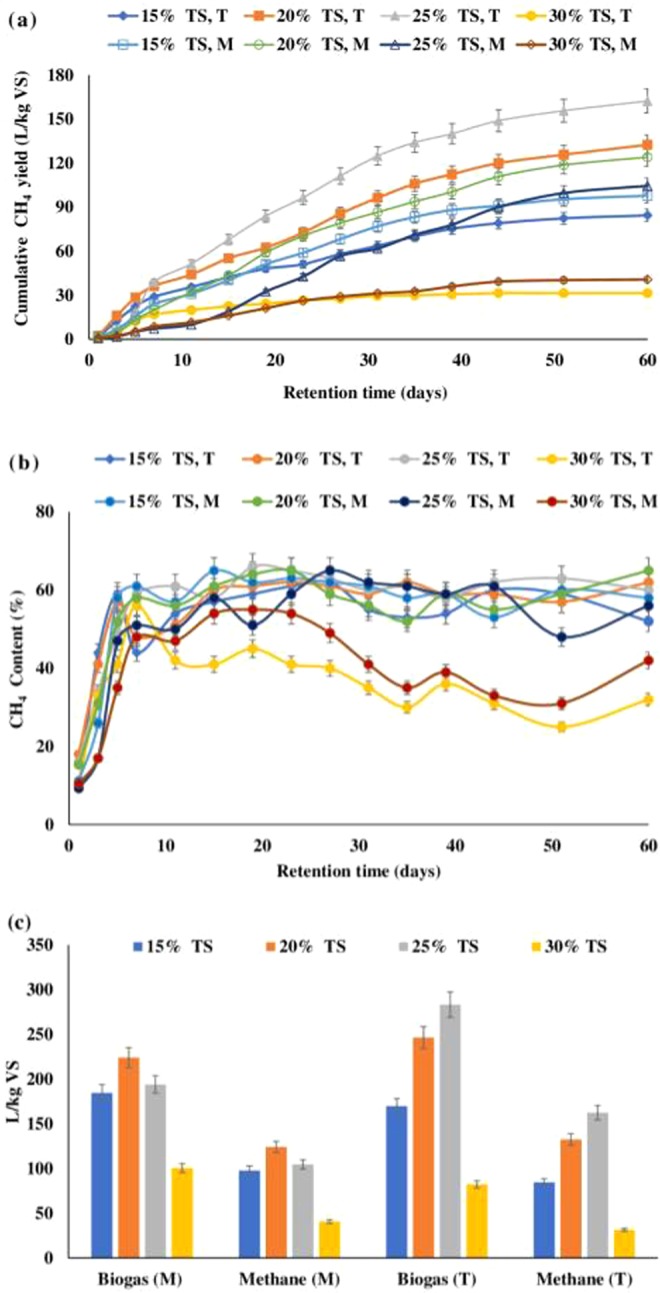
SSAD reactor performance. (**a**) Cumulative methane yield (p < 0.5), (**b**) Methane content (p < 0.5) and (**c**) Comparison of biogas and methane yield at mesophilic and thermophilic temperature (p < 0.5). M – Mesophilic (37 °C); T – Thermophilic (55 °C).

As per experimental results, thermophilic condition showed improvement in methane yield which was 30% higher as compared to the best performer at mesophilic temperature. The batch bioreactor having 20 and 25% TS content showed maximum methane production (124.1 ± 7 and 162.4 ± 9L/kg VS respectively; p < 0.05) at mesophilic and thermophilic temperature (Fig. 1) respectively. This may be ascribed to the fact that at thermophilic temperature, hydrolysis might have been improved which is a rate limiting step during AD (Table 3) and provides a quick start-up to the SSAD reactors^9^. For 25% TS, the hydrolysis rate constant, *k* was 0.0276 d^−1^ at thermophilic and for 25% TS, mesophilic condition it was 0.0318 d^−1^. At mesophilic temperature, cumulative methane yield declined after 20% TS and at thermophilic temperature, yield declined after 25% TS (p < 0.05). However, at 15% TS, mesophilic condition showed 97 ± 5L/kg VS of cumulative methane to that at thermophilic temperature where 84 ± 4L/kg VS of cumulative methane was observed (p < 0.05). This could be ascribed to the fact that between 23^rd^ to 35^th^ day, daily methane yield at mesophilic condition were more than that of thermophilic condition. This led to lower cumulative methane yield in the case of 15% TS at thermophilic condition. Also, the hydrolysis rate constant at 15% TS (55 °C) was 0.0406 d^−1^ and for 15% TS (37 °C) it was *k* = 0.0398 d^−1^.

**Table 3:** Rate constant obtained from first order kinetic model.

	15% TS, M	20% TS, M	25% TS, M	30% TS, M	15% TS, T	20% TS, T	25% TS, T	30% TS, T
k	0.0398	0.0349	0.0318	0.0571	0.0406	0.0257	0.0276	0.0862
R^2^	0.9925	0.9932	0.9474	0.9676	0.9871	0.9921	0.9947	0.9840

M – Mesophilic; T – Thermophilic.

Apart from these, major decline was observed in both methane yield and percentage when the TS content was 30% in mesophilic and thermophilic condition (*k* = 0.0571 d^−1^ and 0.0862 d^−1^ respectively) (p < 0.05). The cumulative methane yield was observed to be 40.7 ± 2L/kg VS in mesophilic region and 31.4 ± 2L/kg VS in thermophilic region (Fig. 1). The cumulative methane generated was nearly 3 and 4-fold down as compared to methane yield at 20 and 25% TS at 37 and 55 °C respectively. This could be ascribed to the reason that enhanced TS content (30% TS) leads to decline in pH due to VFA accumulation and consequently a decrease in methanogenesis^18,19^.

### Reactor characteristics

**Figure 2:**
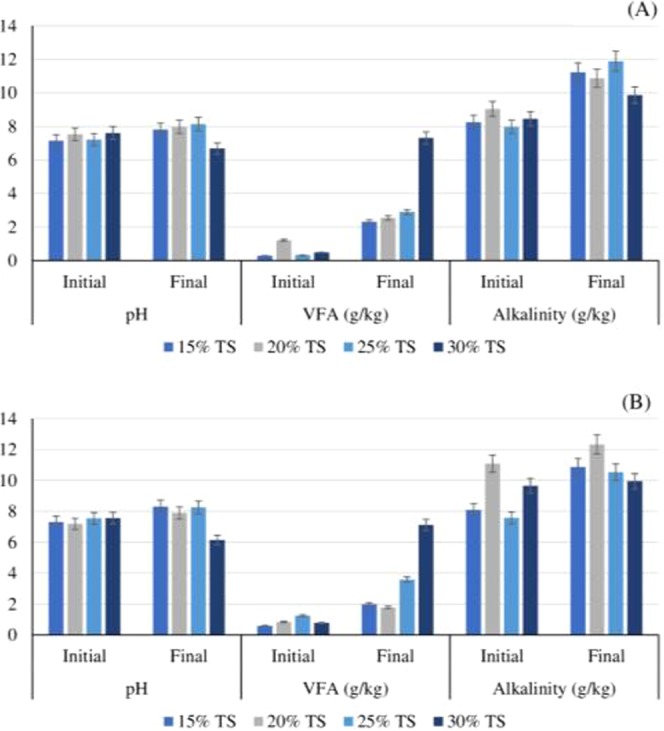
Reactor characteristics (p < 0.05) before and after 60 days of SSAD of PMS. (A – Mesophilic; B – Thermophilic).

Synergistic imbalance between hydrolytic, fermentative, acetogenic and fermentative microflora may disturb the overall SSAD reactor performance and may lower the biogas and methane production^4,9^. VFA accumulation in the reactor which are intermediates in SSAD may cause a dramatic drop of pH, later inhibiting methanogenic microorganism and disrupting the reactor performance of AD. pH is considered to be common stress indicator for monitoring the AD performance^3^. Apart from determining pH value, VFAs and alkalinity were also measured (Fig. 2) as pH is not a sole indicator to assess reactor performance^20^. The initial pH value of all the reactors were ranged between 7.15 to 7.61 and the operational pH was recommended to be 7.4^20^. Figure 2 shows the pH of reactor after the SSAD. A pH drop of 1 unit was observed at 30% TS content at both the operating temperature (37 and 55 °C). This TS content (30%) was also observed to be low in methane productivity (Fig. 1). Also, the VFA was more than 7 g/kg at 30% TS content.

**Figure 3:**
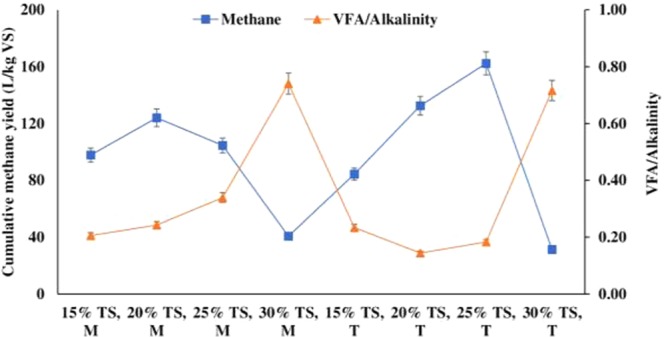
Spectrum of (**a**) Cumulative methane yield and (**b**) VFA/alkalinity ratio (P < 0.5)

On the other hand, alkalinity helps in maintaining the pH of reactor and VFA to alkalinity ratio is a reliable parameter for digester health and should be below 0.6. Once this ratio exceeds the value of 0.6, it may be concluded that SSAD reactor was fed with excessive feedstock^21,22^. Figure 3 shows the spectrum of methane yield and VFA to alkalinity ratio. It was clear that when the VFA to alkalinity ratio is higher than 0.6, the methane yield drops rapidly. This may be ascribed to the fact that accumulation of VFA lowers the pH and interrupts the methanogenic flora causing reduced methane production^23^.

### Cellulose, hemicellulose and VS degradation

Sequential extraction and weighing test method were applied to evaluate the changes in chemical composition of PMS both before and after the SSAD (Table 4). Thermophilic temperature showed better organic matter removal. At thermophilic temperature, cellulose removal was 10, 14 and 10% higher to that mesophilic temperature for TS% 15, 20 and 25 respectively. SSAD of PMS at mesophilic and thermophilic temperature also showed a greater removal efficiency of hemicellulose. Highest hemicellulose removal was observed to be 54.5% for the TS content of 25% at 55 °C followed by 20% TS content (51.5%) at same temperature. Thus, it could be inferred that methane may have been produced from cellulose and hemicellulose degradation. It was observed that cellulose and hemicellulose degradation was higher at thermophilic temperature. This could be explained by the fact that thermophilic temperature provided a rapid start to the reactor and utilized biodegradable waste much efficiently compared to mesophilic temperature^24^. However, TS content of 30% showed 13% (*p* < 0.05) lesser consumption as compared to 25% TS of cellulose at thermophilic temperature (Table 4). This could be ascribed to the fact that increased TS content at thermophilic temperature inhibits methanogenesis and high solid concentration results in VFA accumulation and increase VFA to alkalinity ratio^9,24^.

**Table 4:** Cellulose, hemicellulose and VS removal (p < 0.05) after 60 days of SSAD.

Temperature	TS (%)	% Removal	Hemicellulose	VS
		Cellulose		Degradation
Mesophilic	15	26.3 ± 4.1	32.0 ± 2.9	35.5 ± 2.2
	20	25.5 ± 3.2	33.8 ± 1.8	37.8 ± 3.5
	25	27.1 ± 3.9	31.4 ± 3.3	36.5 ± 1.7
	30	17.0 ± 1.6	22.7 ± 0.9	28.9 ± 0.7
Thermophilic	15	36.8 ± 6.8	46.3 ± 5.4	33.5 ± 3.1
	20	39.1 ± 5.9	51.5 ± 4.6	38.5 ± 2.9
	25	37.9 ± 2.8	54.5 ± 3.9	39.9 ± 2.7
	30	14.2 ± 1.8	22.9 ± 1.1	26.4 ± 1.2

**Figure 4:**
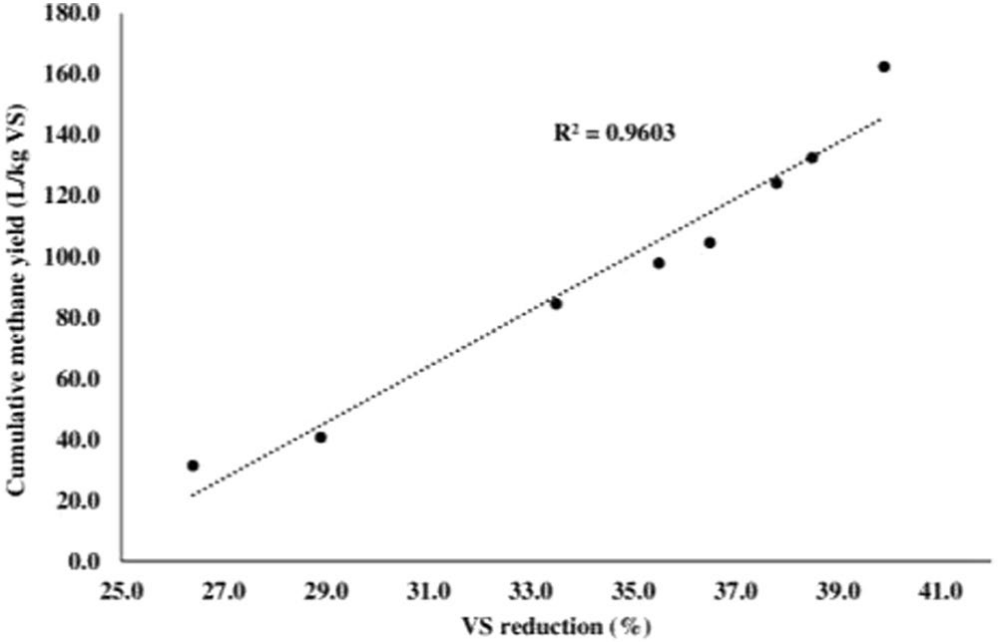
Correlation between cumulative methane yield and VS reduction.

At mesophilic temperature, 20% TS content showed maximum VS reduction (37.8 ± 3.5%) while at thermophilic temperature, 25% TS content was observed to have maximum degraded VS (39.9 ± 2.7%). It was noteworthy that despite of increasing the TS content, no positive effect on VS consumption was observed. At both the temperatures (37 and 55 °C), 30% TS content showed only 22.7 ± 0.9 and 22.9 ± 1.1% VS reduction respectively which was around 15 and 17% lesser to that optimum one. Similar trend was shown in by Sheets *et al*.^11^ in which authors performed SSAD of switchgrass at 20 and 30% TS. The research group observed that VS removal in SSAD of switchgrass was 4 to 5% less at both mesophilic and thermophilic temperature for 30% TS. This clearly showed that for SSAD, desirable TS content for lignocellulosic stubble and waste may be between 20 to 25%. Also, positive correlation (R^2^ = 0.9603) was observed between VS reduction and methane yield and VS reduction (Fig. 4). Brown and Li^25^, and Li *et al*.^26^, also correlated the VS reduction with methane yield for batch SSAD and observed positive results.

### Multicriteria decision making modelling (MCDM)

After completing the biomethane potential test and analytical analysis, lots of experimental data were generated. Possible alternatives (TS content and digestion temperature), experimental and analytical data (best possible output) were arranged in a decision matrix form (Table 5) to employ VIKOR and TOPSIS methods. After creating the decision matrix, normalized matrices were created to make every output dimensionless (Tables 6 and 7) for VIKOR and TOPSIS method using Equations 1 and 9 respectively. In VIKOR method, linear normalization used whereas for TOPSIS, vector normalization was applied. After generating normalized matrix for VIKOR analysis, Value of entropy (*E*_*j*_), dispersion (*π*_*j*_), weight (*ω*_*j*_), Utility measure (*α*_*i*_), regret measure (*β*_*i*_), VIKOR index (Ω_*j*_) and rank of each alternative were determined by using Equations 2, 3, 4, 5, 6, 7 and 8 respectively (Tables 8 and 9). For TOPSIS, closeness index (CI) was determined and rank of the alternatives were obtained (Table 10) using Equations 10, 11 and 12.

**Table 5:** Decision matrix as per experimental results of SSAD of PMS.

Alternatives	VS reduction (%)	VFA (g/kg)	Alkalinity (g/kg)	VFA/alkalinity	Cellulose removal (%)	Hemicellulose removal (%)	CH_4_ yield (L/kg VS)
15% TS, M	35.5	2.3	11.2	0.2	26.3	32.0	97.8
20% TS, M	37.8	2.5	10.9	0.2	25.5	33.8	124.1
25% TS, M	36.5	2.9	11.9	0.2	27.1	31.4	104.6
30% TS, M	28.9	7.3	9.9	0.7	17.0	22.7	40.7
15% TS, T	33.5	2.0	10.9	0.2	36.8	46.3	84.4
20% TS, T	38.5	1.8	12.3	0.1	39.1	51.5	132.5
25% TS, T	39.9	3.6	10.5	0.3	37.9	54.5	162.4
30% TS, T	26.4	7.1	9.9	0.7	14.2	22.9	31.4

**Table 6:** Normalized matrix for each alternatives and criterion for VIKOR.

Alternatives	VS reduction	VFA	Alkalinity	VFA/alkalinity	Cellulose Removal	Hemicellulose Removal	CH_4_ yield
15% TS, M	0.168485999	0.122741764	0.003067388	0.117509042	0.153084983	0.146964121	0.16749305
20% TS, M	0.179401993	0.134962806	0.003484501	0.13348831	0.148428405	0.15524379	0.212402843
25% TS, M	0.173232084	0.153560043	0.003624536	0.138852931	0.15774156	0.144204232	0.178992592
30% TS, M	0.137161841	0.388416578	0.011044261	0.423096358	0.09895227	0.104185833	0.06972461
15% TS, T	0.15899383	0.105738576	0.002729983	0.104583361	0.214202561	0.21274149	0.144529569
20% TS, T	0.182724252	0.094580234	0.002152749	0.082469999	0.227590221	0.236660534	0.226857336
25% TS, T	0.189368771	0.190223167	0.005064993	0.194035627	0.220605355	0.250459982	0.277926692
30% TS, T	0.12529663	0.378320935	0.010681446	0.409197217	0.082654249	0.105105796	0.053783708

**Table 7:** The normalized decision matrix for TOPSIS method.

Alternatives	VS reduction	VFA	Alkalinity	VFA/alkalinity	Cellulose Removal	Hemicellulose Removal	CH_4_ yield
15% TS, M	0.359522107	0.192356	0.361855428	0.174639458	0.316926131	0.292531396	0.326877012
20% TS, M	0.382815088	0.211509	0.350255432	0.198387509	0.307285793	0.309012038	0.414522313
25% TS, M	0.36964949	0.240654	0.383122087	0.206360295	0.32656647	0.287037849	0.349319351
30% TS, M	0.292681377	0.608712	0.318033221	0.628796877	0.204857195	0.207381412	0.13607354
15% TS, T	0.33926734	0.16571	0.350255432	0.155429584	0.443455575	0.423460941	0.282061814
20% TS, T	0.389904256	0.148223	0.39729986	0.122565172	0.471171549	0.471071685	0.442731493
25% TS, T	0.404082593	0.298111	0.339622102	0.288371653	0.456711041	0.498539422	0.542397709
30% TS, T	0.267362919	0.59289	0.320288776	0.608140268	0.17111601	0.209212595	0.104963507

**Table 8:** Value of entropy, dispersion and weight of each alternative for VIKOR.

Alternatives	Entropy, *E*_*j*_	Dispersion, *π*_*j*_	Weight, *ω*_*j*_
15% TS, M	0.815262855	0.184737145	0.14996786
20% TS, M	0.850451931	0.149548069	0.121401702
25% TS, M	0.84851746	0.15148254	0.122972087
30% TS, M	0.819305166	0.180694834	0.14668635
15% TS, T	0.827650417	0.172349583	0.139911755
20% TS, T	0.84980314	0.15019686	0.121928384
25% TS, T	0.967455198	0.032544802	0.026419561
30% TS, T	0.789708918	0.210291082	0.170712302

**Table 9:** Utility measure (*α*_*i*_), regret measure (*β*_*i*_), VIKOR index (Ω_*i*_) and rank of each alternative.

Alternative	Utility measure (*α*_*i*_)	regret measure (*β*_*i*_)	VIKOR index (Ω_*i*_)	Rank
15% TS, M	0.643162549	0.13559481	0.749019116	6
20% TS, M	0.479694555	0.1047172	0.545493493	4
25% TS, M	0.456742217	0.10261686	0.524294003	3
30% TS, M	0.679340522	0.14668635	0.808115856	7
15% TS, T	0.547061916	0.13459865	0.685995198	5
20% TS, T	0.29578437	0.121928384	0.487982168	2
25% TS, T	0.056083628	0.01922399	0	1
30% TS, T	0.860461381	0.170712302	1	8

**Table 10:** The closeness index and the ranking of the alternative.

Alternative	${{\boldsymbol{D}}}_{{\boldsymbol{i}}}^{+}$	${{\boldsymbol{D}}}_{{\boldsymbol{i}}}^{-}$	CI	Rank
15% TS, M	8.690777957	0.793682	0.083682394	4
20% TS, M	8.638189905	0.767816	0.081630396	5
25% TS, M	8.743345793	0.751841	0.079181254	6
30% TS, M	8.470125071	1.011437	0.106674054	2
15% TS, T	8.752018133	0.748764	0.078810783	7
20% TS, T	8.7346088	0.735164	0.077632742	8
25% TS, T	8.505925721	0.785713	0.084561318	3
30% TS, T	8.515281481	1.019374	0.106912541	1

The VIKOR and TOPSIS rank secured by each alternative showing the effect of TS and temperature conditions on the performance of the bioreactor by considering every output into the equation were shown in Tables 8 and 10 respectively. The bioreactor with 25% TS at mesophilic condition and the bioreactor with 20% TS at thermophilic condition obtained first and second rank respectively by VIKOR method and secured third and eighth rank as per TOPSIS method. However, the experimental results were in agreement with the ranking provided by VIKOR method in which 25% TS content showed maximum cumulative methane yield at thermophilic temperature. Also, as per VIKOR ranking, third and fourth rank was obtained by the 25% TS and 20% TS at mesophilic temperature. All other alternatives with TS content other than 20 and 25% at mesophilic and thermophilic temperatures respectively showed least favoured rank (5–8) by VIKOR method. This clearly shows that SSAD of PMS is favoured with TS content ranging between 20 to 25%.

Both the MCDM approaches provide a list of ranking of the alternatives. The alternative which obtained the highest rank by the VIKOR method shows closeness to the ideal solution while the highest rank secured by alternative by TOPSIS method shows the best one in terms of ranking index^27^. Moreover, the rank provided to the alternatives by TOPSIS may not close to the ideal solution^27^ and experimental results were validating this statement. The ranking comparison of alternatives with TOPSIS and VIKOR revealed that VIKOR method is preferred over TOSIS for ranking of TS content and temperature preference for SSAD.

## Methods

### Biomass feedstock and seed inoculum

Pearl millet (*Pennisetum glaucum*) straw (PMS) was collected from Jaipur (26.8°N, 75.9°E) in the month of March 2017. Once collected, the straw was dried and stored in an air tight container prior to the experiment. Seed inoculum was collected for BMP assay from a local biogas plant (active), Jaipur (26.8°N, 75.7°E), India. The biogas plant was fed with cow dung and have a continuous stirred-type bioreactor design operating at mesophilic temperature. The seed inoculum was pre-incubated anaerobically for 5 days to reduce the endogenous gas production.

### Solid state anaerobic digestion

The effect of different solid concentrations (15, 20, 25 and 30%) of PMS on mesophilic (37 °C) and thermophilic (55 °C) temperature were studied for biomethanation. The PMS was shredded and pre-mixed manually with active inoculum to achieve feedstock/inoculum (F/I) ratio of 1 (on VS basis) for all the solid concentration in accordance with prior results^28,29^. The premixed feedstock was filled in anaerobic glass bottles (610 mL) in triplicates and sealed with rubber septum and aluminium screw cap along with inoculum without any feedstock as negative controls. Deionized water was added into each bottle to adjust the solid content from 15 to 30%. All the batch bioreactors were placed in incubators (REMI CIS 24, India) at mesophilic and thermophilic conditions (37 and 55 °C) for 60 days and prior to incubation all reactors were purged with nitrogen to create anaerobic condition. Manual mixing of anaerobic bioreactors was performed twice a day by tilting them upside down without opening the rubber stoppers. Biogas composition analysis and calculation was performed as described previously^2,30^. In short, the pressure was measured by a digital pressure meter (Testo 512, Germany) and the biogas composition was determined by gas chromatogram (TRACE 1300, Thermo Fisher Scientific, India) equipped with thermal conductivity detector (TCD) and Helium as carrier gas. All the batch reactors were purged after volume calculation with needle.

### Analytical methods

Characterization and Compositional analysis of PMS was performed both before and after the digestion period. The TS, VS, pH and alkalinity content were determined as per American public health association (APHA) guidlines^31^. Ultimate analysis (C, H and N) was performed using Elemental Analyzer (FLASH 2000; Thermo Scientific, USA). Hot water extractives, cellulose, hemicellulose and lignin present in PMS was calculated by sequential extraction and weighing method^32^. Hot water-soluble materials was determined by dissolving the straw samples in 75 ml water by boiling for 1 hr and after 1 hr, fresh water added to replace former hot water and again boiled for 1 hr. Cold water was used to wash samples after boiling, and dried overnight at 60 °C for 15 h and weighed. Dried sample was then dissolved in 30 ml water with 2 ml 10% acetic acid and 0.6 g Sodium Chlorite followed by heating at 75 °C for 1 h as lignin estimation procedure. After 1 h same procedure has been repeated and heated for another 2 h at 75 °C. After 2 h, washing was carried out with water, acetone and ether (five times, two times and once respectively). After washing, samples were then dried at 105 °C for 90 mins and weighed. After lignin estimation hemicellulose was quantified by adding 24% KOH (20 ml) and left at 20 °C in air. Samples were then washed five times with water, once with 5% acetic acid, once again with water, once with acetone and once with ether. After washing the sample were dried at 105 °C for 90 mins followed by weighing. The residual weight was taken as cellulose. For calculating cellulose, hemicellulose and lignin removal, same procedure was adopted prior to the start of experiment with inoculum and PMS combined and after the end of the experiment i.e. on 60^th^ day.

VFAs was measured by titration methods as per described in previous studies^33^. Samples for measuring VFA was prepared by dissolving 5 g of sample to 50 mL deionized water and filtered through cheese cloth having four layers.

### Multicriteria decision making modeling

In this study TOPSIS and VIKOR technique was applied to get a deep insight of application of MCDM to BMP test. The steps involved in VIKOR and TOPSIS method are described below.

### VIKOR method

Step 1: create a decision matrix of alternative selected for experiment and output.

$${D}_{mn}=[\begin{array}{ccc}{x}_{11} & {x}_{12} & {x}_{1m}\\ {x}_{21} & \ldots  & \ldots \\ {x}_{n1} & \ldots  & \ldots \end{array}]$$

Step 2: Create a normalized matrix using equation

1 $${\rho }_{ij}=\frac{{x}_{ij}}{{\sum }_{i=1}^{n}\,{x}_{ij}}\,$$

Step 3: After creating normalized matrix, find entropy of each alternative

2 $${E}_{j}=\,-\,k\,\mathop{\sum }\limits_{i=1}^{m}\,{\rho }_{ij}\,\mathrm{ln}({\rho }_{ij}),$$

where k = 1/ln (m)

Step 4: Calculate dispersion value of each alternative

3 $${\pi }_{j}=1-{E}_{j}$$

Step 5: Find weight of each alternative

4 $${\omega }_{j}=\frac{{\pi }_{j}}{{\sum }_{j=1}^{n}\,{\pi }_{j}}$$

Step 6: Determine utility measure (*α*_*i*_) and regret measure (*β*_*i*_) using weights of each alternative.

5 $${\alpha }_{i}=\mathop{\sum }\limits_{j=1}^{n}\,{\omega }_{j}\frac{[{x}_{ijmax}-{x}_{ij}]}{[{x}_{ijmax}-{x}_{ijmin}]}\,(if\,j\,is\,benefit\,criteria)\,for\,j=1,\,2\ldots m$$

6 $${\alpha }_{i}=\mathop{\sum }\limits_{j=1}^{n}\,{\omega }_{j}\frac{[{x}_{ij}-{x}_{ijmin}]}{[{x}_{ijmax}-{x}_{ijmin}]}\,(if\,j\,is\,cost\,criteria)\,for\,j=1,2\ldots \ldots .m$$

and

7 $${\beta }_{i}=\,{\rm{\max }}\,of\,\{{\omega }_{j}\frac{[{x}_{ij}-\,{x}_{ijmin}]}{[{x}_{ijmax}-\,{x}_{ijmin}]}\}\,for\,j=1,2,\,\ldots \ldots \ldots \ldots m$$

from decision matrix, obtain maximum (*x*_*ij*_*max*) and minimum (*x*_*ij*_*min*) value for each output.

Step 7: finally calculate VIKOR index, Ω_*i*_

8 $${\Omega }_{i}=\varepsilon (\frac{{\alpha }_{i}-\,{\alpha }_{i}^{-}}{{\alpha }_{i}^{+}-\,{\alpha }_{i}^{-}})+(1-\varepsilon )(\frac{{\beta }_{i}-{\beta }_{i}^{-}}{{\beta }_{i}^{+}-{\beta }_{i}^{-}})$$

where ${\alpha }_{i}^{+}and\,{\beta }_{i}^{+}=$ *max of* *α*_*i*_ *and β* (*i*=1,  2, … .. *m*) and ${\alpha }_{i}^{-}and{\beta }_{i}^{-}=\,{\rm{\min }}\,of\,{\alpha }_{i}\,and$ $\beta (i=1,\,2,\,\ldots \mathrm{..}\,m)$*ε* is introduced as weight for the maximum value of utility and (1 − *ε*) is the weight of the individual regret and normally its value of *ε* is taken as 0.5.

### TOPSIS method

Step 1: create a decision matrix of alternative selected for experiment and output.

$${D}_{mn}=[\begin{array}{ccc}{x}_{11} & {x}_{12} & {x}_{1m}\\ {x}_{21} & \ldots  & \ldots \\ {x}_{n1} & \ldots  & \ldots \end{array}]$$

Step 2: Determine the normalized matrix by calculating normalized value. The normalized value calculated as

9 $${r}_{ij}=\frac{{x}_{ij}}{\sqrt{({\sum }_{i=1}^{n}\,{x}_{ij})\,}}$$

Step 3: Determine the positive ideal solution and the negative ideal solution

10 $${A}^{+}={r}_{1}^{+},\,{r}_{2}^{+},{r}_{3}^{+}\ldots \ldots {r}_{n\,}^{+}$$

and

11 $${A}^{-}={r}_{1}^{-},\,{r}_{2}^{-},{r}_{3}^{-}\ldots \ldots \ldots {r}_{n}^{-}$$

where

$$\begin{array}{lllll}{r}_{ij}^{+}=\tfrac{(ma{x}_{i}{r}_{ij}),\,if\,j\,is\,benefit\,criteria}{(mi{n}_{i}{r}_{ij}),\,if\,j\,is\,cost\,criteria} & and & {r}_{ij}^{-}=\tfrac{(mi{n}_{i}{r}_{ij}),\,if\,j\,is\,benefit\,criteria}{(ma{x}_{i}{r}_{ij}),\,if\,j\,is\,cost\,criteria} & for & j=1,\,2\ldots \ldots \ldots .n\end{array}$$

Step 4: The Euclidian distances between each of the alternatives and the positive ideal solution and the negative ideal solution are calculated as shown

12 $${D}_{i}^{+}=\sqrt{(\mathop{\sum }\limits_{j}^{n}\,{({r}_{ij}^{+}-{r}_{ij})}^{2})}$$

and

13 $${D}_{i}^{-}=\sqrt{(\mathop{\sum }\limits_{j}^{n}\,{({r}_{ij}-{r}_{ij}^{-})}^{2})}$$

Step 5: Finally, determine the overall preference or closeness index (CI) of the alternatives. The closeness index (CI) of the alternatives is calculated as

14 $$C{I}_{i}=\frac{{D}_{i}^{-}}{{D}_{i}^{+}+{D}_{i}^{-}\,}$$

### Statistical analysis

All the data were tested for the level of significance and analysis of variance (ANOVA; p < 0.05) was performed in Microsoft excel spreadsheet (version 2016) using solver function.

### Kinetic study

First order kinetic model^27^ was used to determine the hydrolysis constant for both mesophilic and thermophilic condition. The first order kinetic equation is as below.

15 $${Y}_{t}={Y}_{max}\ast [1-exp(\,-\,kt)]$$

where, *Y*_*t*_ = cumulative methane yield (L/kg VS) at time *t*(*d*); *Y*_*max*_ = maximum cumulative methane production and *k* = hydrolysis constant (d^−1^).

## Conclusions

TS and temperature play vital role in SSAD of PMS. With 25% TS, PMS may be digested anaerobically at thermophilic temperature for higher methane yield (1.3 folds) as compared to mesophilic. There is an upper limit of TS content at both mesophilic and thermophilic temperature and beyond which VFA accumulation and decreased methane yield may be observed. VFA, pH and alkalinity showed the performance of SSAD reactor over the digestion period. Also, VFA to alkalinity ratio may be validated with every reactor output. MCDM approach provided ranking to the alternatives (temperature and TS) for SSAD of PMS. While VIKOR provides ranking considering closeness to the ideal solution, TOPSIS ranks it by selecting alternative which is having shortest distance from ideal solution. TOPSIS considers two reference point for providing ranking and ignores relative importance. So, while the TOPSIS provided an ambiguous ranking considering experimental results, VIKOR method showed agreement with the experimental result of BMP test.
